# Diphthamide-deficiency syndrome: a novel human developmental disorder and ribosomopathy

**DOI:** 10.1038/s41431-020-0668-y

**Published:** 2020-06-23

**Authors:** Harmen Hawer, Bryce A. Mendelsohn, Klaus Mayer, Ann Kung, Amit Malhotra, Sari Tuupanen, Jennifer Schleit, Ulrich Brinkmann, Raffael Schaffrath

**Affiliations:** 1 Fachgebiet Mikrobiologie, Institut für Biologie, Universität Kassel, D-34132 Kassel, Hessen Germany; 2 Kaiser Permanente Oakland Medical Center, Oakland, CA 94611 USA; 3 Roche Pharma Research & Early Development, Large Molecule Research, Roche Innovation Center Munich, D-82377 Penzberg, Bavaria Germany; 4 Blueprint Genetics Oy, Keilaranta 16 A-B, 02150 Espoo, Finland; 5 Blueprint Genetics, Seattle, WA 98121 USA

**Keywords:** Genetics, Disease genetics

## Abstract

We describe a novel type of ribosomopathy that is defined by deficiency in diphthamidylation of translation elongation factor 2. The ribosomopathy was identified by correlating phenotypes and biochemical properties of previously described patients with diphthamide biosynthesis gene 1 (*DPH1*) deficiencies with a new patient that carried inactivating mutations in both alleles of the human diphthamide biosynthesis gene 2 (*DPH2*). The human *DPH1* syndrome is an autosomal recessive disorder associated with developmental delay, abnormal head circumference (microcephaly or macrocephaly), short stature, and congenital heart disease. It is defined by variants with reduced functionality of the *DPH1* gene observed so far predominantly in consanguineous homozygous patients carrying identical mutant alleles of *DPH1*. Here we report a child with a very similar phenotype carrying biallelic variants of the human *DPH2*. The gene products DPH1 and DPH2 are components of a heterodimeric enzyme complex that mediates the first step of the posttranslational diphthamide modification on the nonredundant eukaryotic translation elongation factor 2 (eEF2). Diphthamide deficiency was shown to reduce the accuracy of ribosomal protein biosynthesis. Both DPH2 variants described here severely impair diphthamide biosynthesis as demonstrated in human and yeast cells. This is the first report of a patient carrying compound heterozygous DPH2 loss-of-function variants with a *DPH1* syndrome-like phenotype and implicates diphthamide deficiency as the root cause of this patient’s clinical phenotype as well as of *DPH1*-syndrome. These findings define “diphthamide-deficiency syndrome” as a special ribosomopathy due to reduced functionality of components of the cellular machinery for eEF2-diphthamide synthesis.

## Introduction

Diphthamide on eukaryotic elongation factor 2 (eEF2) is a highly conserved posttranslational modification [1]. While eEF2 function for ribosomal translocation is essential for mRNA translation and de novo protein synthesis, the physiological function of the diphthamide moiety is not fully understood [2]. Structural analysis of eEF2 inside the ribosome indicates that diphthamide may act as a *pawl* linking with both mRNA and rRNA in the decoding center averting reading frame errors [3], and using yeast models, diphthamide was assigned a function in the prevention of -1 frameshifting [4–6]. In human cells, diphthamide may modulate translational decisions at paused ribosomes (stall vs. continuation vs. termination). In line with that, loss of diphthamide influences the expression levels of tRNA aminoacyl synthetases and importantly, affects the expression of selenoproteins via reduced selenocysteine incorporation at recoded SECIS-UGA codons [7]. Diphthamide deficiency also correlates with preinduction of stress pathways and gene signatures in mammalian cells, including those associated with oxidative stress responses [7] and nuclear factor of kappa light polypeptide gene enhancer in B cells (NF-κB)—transcript signatures and tumor necrosis factor-mediated pathways [8].

In yeast, diphthamide biosynthesis was elucidated to proceed in four consecutive steps requiring a minimum of seven gene products (Dph1–Dph7) as well as S-adenosyl methionine as an essential co-factor [2]. In the first step, the Dph1–Dph2 heterodimer generates a 3-amino-3-carboxypropyl (ACP)-modified histidine in a process that depends on electron donation by Dph3 and J-type chaperone Dph4 [9, 10]. Next, Dph5 generates methylated diphthine from the ACP-modified intermediate [10] followed by a demethylation reaction catalyzed by Dph7 [11]. Finally, Dph6 mediates the amidation reaction to generate fully diphthamide-modified eEF2 [5].

So far, only one gene involved in diphthamide biosynthesis has been associated with human disease: diphthamide biosynthesis gene 1 (*DPH1*) was originally identified as tumor suppressor gene *OVCA1* with frequent observations of loss of heterozygosity in various cancers, in particular ovarian cancer [12–14]. More recently, loss of *DPH1* function has also been reported as the cause of developmental phenotypes in humans (OMIM #603527, #616901) [13, 15]. Individuals with this disorder, also referred to as *DPH1* syndrome [15], are characterized by varying degrees of intellectual disability. Other findings reported in a majority of cases include central nervous system (CNS) malformations, hypotonia, short stature, abnormal head circumference (either microcephaly or macrocephaly), epilepsy, dental anomalies, unusual skull shape, sparse or high facial or scalp hair, hand/foot anomalies, and abnormal toe nails. Less common findings included cardiac defects, genital anomalies, and renal disease. Functional studies demonstrated a loss-of-function mechanism for this syndrome with autosomal recessive inheritance [15].

Here we present the first case of diphthamide syndrome in a patient from non-consanguineous parents with compound heterozygous diphthamide biosynthesis gene 2 (*DPH2*) variants. We analyzed the catalytic activity of the human DPH2 variants and their yeast homologues, revealing both variants drastically reduce or abolish diphthamide biosynthesis on eEF2.

## Methods

*Whole exome sequencing* (WES) was performed for the index patient and his unaffected parents in a CLIA-certified laboratory (Blueprint Genetics). Informed consent for exome sequencing was obtained from the parents as part of standard clinical care and additional informed consent was obtained to publish medical and genetic findings as well as his presentation to improve the knowledge of rare disease. Total genomic DNA was extracted from blood by standard protocol. The exonic and selected noncoding regions were captured with IDT xGen Exome Research Panel with custom-designed capture probes, followed by paired-end sequencing (150 × 150 bases) using the Illumina sequencing system (NovaSeq). Sequencing-derived raw image files were processed using a base-calling software (Illumina) and the sequence data were transformed into FASTQ format. Clean sequence reads of each sample were mapped to the human reference genome (GRCh37/hg19). Burrows–Wheeler Aligner (BWA-MEM) software was used for read alignment. Duplicate read marking, local realignment around indels, base quality score recalibration, and variant calling were performed using GATK algorithms (Sentieon). The sequencing depth and coverage for the tested sample were calculated based on the alignments. The sequencing run included in-process reference sample for quality control, which passed the thresholds for sensitivity and specificity. Samples from the patient were subjected to thorough quality control measures as well, after which raw sequence reads were processed to identify variants.

The WES covered 99.5%, 99.3%, and 99.5% of the bases at 20× for the index patient, mother, and father, respectively. Identified variants were annotated using Ensembl’s Variant Effect Predictor (VEP v87) tool [16]. High-quality data were filtered for rare coding and intronic (±20 bps from the exon–intron boundaries) variants (≤3 homozygotes in Genome Aggregation Database, gnomAD). The analysis focused on de novo variants and compound heterozygous variants in known human disease genes. Candidate variants in genes where disease association has not yet been established included all coding region de novo variants and rare (<1% MAF in gnomAD) truncating homozygous or compound heterozygous variants, and a rare truncating variant in a compound heterozygous state with a rare missense variant that is predicted deleterious by multiple in silico tools. SIFT, PolyPhen, and MutationTaster pathogenicity scores and predictions were obtained for each missense variant. The DPH2 variant nomenclature is based on the accession number NM_001384.4 from the RefSeq database [17]. Variants analyzed in this work were submitted to the ClinVar repository (SUB7241952).

*Structure models* of human and *Saccharomyces cerevisiae* DPH2 proteins were generated with Phyre2 web portal [18] using *Pyrococcus horikoshii* DPH2, PDB:3LZD [19], as a template. Visualization of the resulting models was performed using PyMol 2.3 (Version 2.3 Schrödinger, LLC, New York, NY, USA). Important residues conserved from yeast to men were identified via sequence alignment using protein BLAST with standard algorithm parameters and human DPH2 as query and *S. cerevisiae* Dph2 as subject. All analyses directed at yeast DPH2 applied the *S. cerevisiae* S288C reference genome (R64/sacCer3) with the RefSeq database accession number GCF_000146045.2 [17].

*Generation of genomic DPH2 point mutations* in yeast was performed utilizing the *delitto perfetto* approach [20]. PCR-based counterselectable reporter (CORE) cassettes were transformed in BY4741 cells to disrupt *DPH2* using standard lithium acetate transformation and selection on minimal (SD) medium lacking uracil. Correct insertion of the CORE cassette was verified using diagnostic PCR. Site-directed mutagenesis PCR was performed on *DPH2* fragments inserted in a pJET1.2 cloning vector (Thermo Fisher scientific; Cat. No. 1231) and the resulting mutant variants were amplified via PCR and transformed in cells with CORE-based *DPH2* disruption. After recovery on yeast extract peptone dextrose (YPD) plates at 30 °C for 24 h, counterselection was performed by growing cells in liquid minimal (SD) medium containing 1 g/L 5-FOA (5′-fluorootic acid) at 30 °C for 6 h and plating 200 µl of the liquid culture on SD-plates containing 5-FOA (1 g/L). Resulting mutant cells were verified using standard colony PCR protocols and sequencing PCR products amplified from genomic DNA preparations.

*Detection of unmodified eEF2 by Western blots* was achieved by preparing total cell extracts of BY4741 wild type and mutant yeast cells from cultures inoculated in YPD medium at 30 °C at OD_600_ nm of 0.1 and harvested at OD_600_ nm of 1. Cells were disrupted via glass bead lysis [21] and protein concentrations were normalized using standard Bradford assays [22] before denaturing samples in SDS buffer [23]. Protein extracts were run on reducing SDS-PAGE and blotted on PVDF membranes (Merck/Millipore; Cat. No. IPVH07850) using a semi-dry transfer system (Biorad; Cat. No. 1704150). Detection of total and unmodified eEF2 was performed using *anti-eEF2 (pan)* (3C2) (1:5000) and *anti-eEF2 (no diphthamide)* (10G8) (1:15,000) antibodies [8], both of which were previously shown to detect modified and unmodified yeast eEF2 [6]. *Anti-Cdc19* antibodies (1:400,000) (a kind gift from Jeremy Thorner, University of California, Berkeley, USA) were used to detect Cdc19 (cell division cycle 19, a pyruvate kinase and housekeeping gene in yeast) to ensure equal loading.

*Viability assays of wild type and DPH2 mutant yeast derivatives* were performed by spotting tenfold cell dilutions starting at OD_600_ nm of 1.5 on full YPD medium containing 10 or 12.5 µg/µl sordarin or 30, 40, 50, 60, or 80 µg/µl hygromycin B, respectively. A plasmid carrying the diphtheria toxin (DT) ADP-ribosylation (ADPR) domain fused to a galactose-inducible promoter (pSU9) [5] was transformed in wild type and *DPH2* mutant variants to assess intracellular activity of the toxin. For the DT assay, spots were generated using fivefold serial cell dilutions starting at OD_600_ nm of 0.5 on minimal (SD) medium lacking uracil and containing glucose (2%, DT off) or galactose (2%, DT on) essentially as described [5]. All plates were incubated for 2–3 days at 30 °C.

*ADPR of human diphthamide-modified eEF2* was evaluated via toxin-mediated transfer of biotinylated ADP to eEF2 present in total cell extracts of transfected or non-transfected MCF7 *DPH2*ko cells. The assays were established based on the principle previously described for the analyses of DPH1 and DPH5 variants [24] except that *DPH2*-deficient cells and *DPH2* expression plasmids were applied instead of *DPH1*ko or *DPH5*ko cells and corresponding *DPH1/5* plasmids. Radioimmunoprecipitation assay buffer (RIPA) extracts of *DPH2*ko cells [8] were exposed to DT and biotinylated NAD 1 h at 25 °C, subsequently subjected to reducing SDS-PAGE and blotted onto membranes. Presence of bio-eEF2 (reflects presence of diphthamide) generates a 100 kDa protein signal after probing with streptavidin-HRP and peroxidase substrate.

## Results

### Novel DPH2 variants in a patient with clinical diphthamide syndrome

The patient, a 19-month-old male when last evaluated, was born at 39 weeks gestation via spontaneous vaginal delivery to a 30-year-old mother. The mother had experienced infertility and was treated with letrozole prior to the pregnancy. The birthweight, length, and head circumference were normal. The proband had no siblings. His parents were healthy without developmental concerns in childhood. No first- or second-degree relatives were known to have developmental delays or congenital anomalies. The family ancestry was non-Ashkenazi Jewish Eastern European (mother) and Hispanic, European, and Native American (father). There was no known consanguinity.

**Fig. 1:**
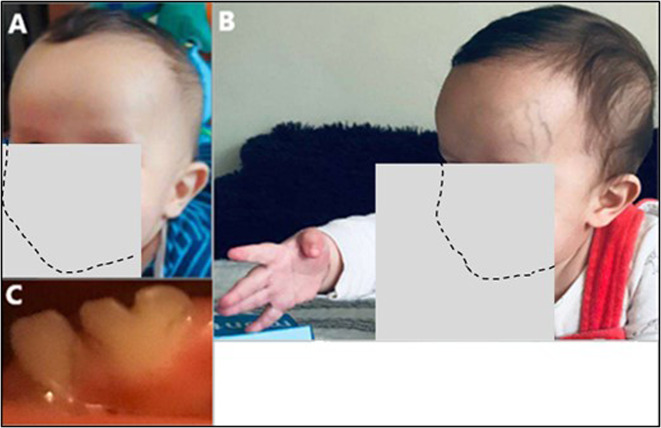
Clinical presentation of the patient. **a** Image at 5 months and **b** at 8 months, note the prominent forehead, high hairline, sparse scalp hair, and single palmar crease on the right hand. **c** Notched primary lower incisor at 14 months. Images were partially obscured to maximize privacy for the patient while highlighting pertinent clinical findings.

Important anomalies include macrocephaly, developmental delays, short stature, dysmorphic craniofacial features, and congenital heart disease. Macrocephaly was appreciated first at 6 months 3 weeks, with a head circumference of 46.5 cm (98.57 percentile) and at 17 months 3 weeks was 50 cm (97.72 percentile). A head ultrasound at 6 months of age showed no anomalies. Magnetic resonance neuroimaging has not been pursued. Developmental delays were noted around 12 months of age and he was referred to genetics and child neurology. At that time, he was able to sit unassisted but could not crawl, pull to stand, and did not wave nor point with one finger to indicate desire. Physical therapy evaluation demonstrated mild gross motor delay and fine motor delays were noted by an occupational therapist. At 19 months, he was able to pull to stand but was not able to walk independently. Also, at that time a formal language assessment placed his expressive language abilities at 14 months. Short stature became apparent by 12 months of age, when the length was 70 cm (0.61 percentile), and at 17 months 3 weeks was 76.2 cm (1.38 percentile). In comparison, the parents are of normal height: the mother is 163 cm and the father is 178 cm tall. Weight and body mass index were always in the normal range. Other structural anomalies include bilateral hydroceles that were first noted at 4 months of age; ultrasound imaging determined that there was no inguinal hernia. A cardiac murmur was detected in the newborn period and an echocardiogram revealed a small peri-membranous ventricular septal defect with tricuspid tissue involvement, with a second small mid-muscular ventricular septal defect. A dysmorphology examination at 12 months was notable for a prominent forehead with visible veins, and a high anterior hairline and spare scalp hair. The ears were noted to be borderline low-set and mildly posteriorly rotated. The eyes were deep-set, related to the prominent forehead, and the right hand had a single-transverse palmar crease (Fig. 1a, b). The thumbs were adducted and infolded bilaterally. Brachydactyly of the fingers and toes was noted; the 3rd finger was 3.1 cm (<3 percentile). The skin was soft but not hyperelastic nor translucent. The hydroceles were still present. When primary dentition erupted, at least one notched tooth was noted (Fig. 1c).

At 12 months of age, trio-based exome sequencing was pursued given the combination of developmental delay, congenital heart disease, and other dysmorphic features. Two candidate variants in trans in the gene *DPH2* were reported: c.922C > T (p.(Gln308*)) paternally inherited, and c.601C > T (p.(Arg201Cys)) maternally inherited. The nonsense variant results in a premature stop codon at Q308 instead of codon 490 as in the wild-type transcript. The missense variant c.601C > T (p.(Arg201Cys)) is predicted deleterious by SIFT, PolyPhen, and MutationTaster in silico tools. No other variants were reported. Both variants are reported in three heterozygous individuals in gnomAD [25]. No reported homozygotes for a loss-of-function *DPH2* variant are described. Significant phenotypic overlap was noted between this patient and reported phenotypes with *DPH1*-related syndrome (Table 1). Furthermore, a 2016 report [26] described two siblings who also had intellectual disability and physical findings similar to those described with reduced function of *DPH1* (short stature, prominent forehead, sparse hair, low-set ears, see Table 1). Those siblings were homozygous for a *DPH2* variant with a (short) C-terminal truncation (c.1429C > T, p.(R477*)), simultaneously carrying a homozygous variant in the unrelated *KALRN* gene. Their phenotypes were proposed to be related to the *KALRN* variant as more was known about the expression of that gene at the time.

**Table 1:** Comparison between patients previously described with diphthamide syndrome related to mutations in *DPH1* (Urreizti et al.) [15], the case related to *DPH2* described in this report, and a case report of siblings homozygous for a truncating *DPH2* variant p.(Arg477*) (Makrythanasis et al.) [26].

Phenotype	Urreizti et al. [15] (*DPH1*)	Present case (*DPH2*) at 19 months of age	Makrythanasis et al. [26] (DPH2 siblings)
Developmental delay/intellectual disability	17/17	Gross motor delay, not walking, fine motor and expressive language delays	Gross motor delay; intellectual disability
CNS malformations	9/11	Not determined	Not present
Epilepsy	5/7	Not present	Not discussed
Short stature	14/15	Length < 2nd percentile	Short stature
Abnormal head circumference	6/9	Macrocephaly, >97 percentile	Microcephaly
Dental abnormalities	7/10	Notched tooth	Not discussed
Sparse hair on scalp	15/15	High hairline, sparse scalp hair	Sparse hair
Dysmorphic features	17/17	Low-set ears	Low-set ears
Hand/foot anomalies	12/12	Brachydactyly, right single-transverse crease	Abnormal palmar creases
Heart malformation	7/15	Ventricular septal defects	Not discussed
Unusual skull shape	15/15	Prominent forehead	High prominent forehead
Abnormal toe nails	6/8	Not present	Not discussed
Genitalia anomalies	5/9	Large bilateral hydroceles	Not discussed

### The c.922C > T (p.(Gln308*)) and c.601C > T (p.(Arg201Cys)) variants are located in functionally relevant regions of *DPH2*

**Fig. 2:**
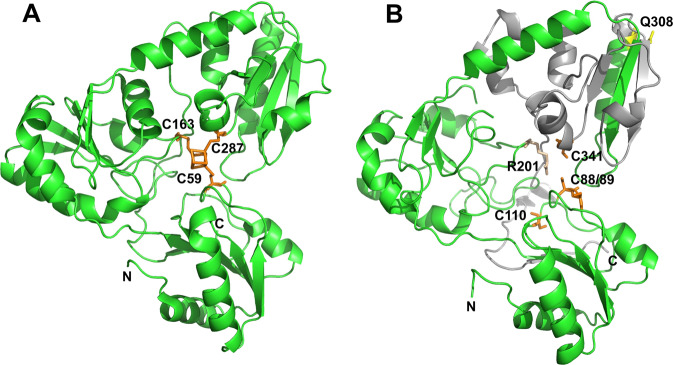
Structural comparison of *Pyrococcus* Dph2 and human DPH2. **a** Structure of the *Pyrococcus horikoshii*. Dph2 is described as a homodimer. To facilitate the structural comparison and localization of the variants, only one monomer of that structure is shown. Cys residues (C59, C163, C287) that carry a [4Fe–4S] cluster essential for catalytic activity (PDB:3LZD [19]) are shown in orange. **b** Model of human DPH2. Structure alignments identify Cys residues (orange) (C88/89 and C341) homologous to cysteines (C59 and C287) that bind the [4Fe–4S] cluster in archaeal Dph2. An additional cysteine is located at position 110. Mutated residues found in the patient (c.922 C > T (p.(Gln308*)) and c.601 C > T (p.(Arg201Cys))) are highlighted in yellow and wheat, respectively. The c.601 C > T (p.(Arg201Cys)) variant is located close to the catalytical center. The region predicted to be absent in the c.922 C > T (p.(Gln308*)) variant is shown in gray. This truncation leads to the absence of a large portion of the catalytical center including C341 and may additionally destabilize or misfold the protein.

Currently, a crystal structure of huDPH2 is not available. However, the sequences of the human DPH1 and DPH2 proteins are conserved in large stretches between each other, as well as among other eukaryotes and archaea. It is therefore possible to define the positions/regions within DPH2 of Gln308 and Arg201 via homology alignments to the sequence of Dph2 from the archaeon *Pyrococcus horikoshii* for which a structure exists (PDB:3LZD) [19]. Some residues and regions of Dph2 that are catalytically important have already been defined in Dph2 from *Pyrococcus* and yeast [19, 27]. Specifically, archaeal Dph2 carries three cysteines (C59, C163, and C287; Fig. 2a) that coordinate an iron–sulfur cluster [19] and recently, a similar iron–sulfur cluster binding domain consisting of three conserved Cys residues (C106, C107, and C362) was reported for yeast Dph2 [27]. These sites have been mapped to the *Pyrococcus* structure (PDB:3LZD) [19], and by sequence homology, can also be assigned to human DPH2 (Fig. 2b). Using this model, we also mapped the positions corresponding to the c.922C > T (p.(Gln308*)) and c.601C > T (p.(Arg201Cys)) variants found in the patient. Figure 2b highlights the positions where changes on the protein level can be expected according to sequence and structure homologies. The c.922C > T (p.(Gln308*)) truncation variant eliminates a large C-terminal portion of the protein (Fig. 2b) including regions expected to be crucial for activity. c.601C > T (p.(Arg201Cys)) substitution introduces an additional cysteine in close proximity to the active center (Fig. 2b). Because this region is defined by and requires an exact reactive cysteine motif by itself [19, 27], incorporation of an additional reactive thiol group into that vicinity is likely to disturb the active center. Furthermore, both variants may disturb the protein structure, folding, or stability and hence may affect the overall levels of DPH2 protein harboring those variants. This in turn may also contribute to or aggravate reduced functionality.

### Strongly reduced diphthamide synthesis activity in the novel human DPH2 variants

The diphthamide modification is the molecular target of bacterial toxins including *Pseudomonas* exotoxin A, DT, and cholix toxin [1, 2]. These toxins inactivate eEF2 by ADPR at the diphthamide residue. In contrast, eEF2 without diphthamide is not subject to ADPR [10, 28]. Therefore, toxin-ADPR assays can be applied to probe the presence or absence of diphthamide on human eEF2. To investigate the effect of the huDPH2 c.922C > T (p.(Gln308*)) and c.601C > T (p.(Arg201Cys)) variants on diphthamide synthesis in human cells using ADPR assays, protein extracts were prepared from DPH2-deficient MCF7 cells recombinantly supplemented with wild-type or mutated *DPH2* genes as previously described [8, 24]. The ADPR reaction transfers ADP to the diphthamide moiety of eEF2, using cell extracts as a source of diphthamide-eEF2 and NAD to donate ADP. Because biotinylated NAD is also accepted as substrate, toxin-mediated ADPR transfers bioADP to diphthamide-eEF2. This places a biotin-label onto eEF2. Presence of bioADP-diphthamide-eEF2 in cell extracts was subsequently monitored by a Western blot procedure, detecting the modified eEF2 via labeled streptavidin [24]. Because the presence of diphthamide is essential for toxin-mediated ADPR, eEF2 that lacks diphthamide does not become labeled via ADPR.

**Fig. 3:**
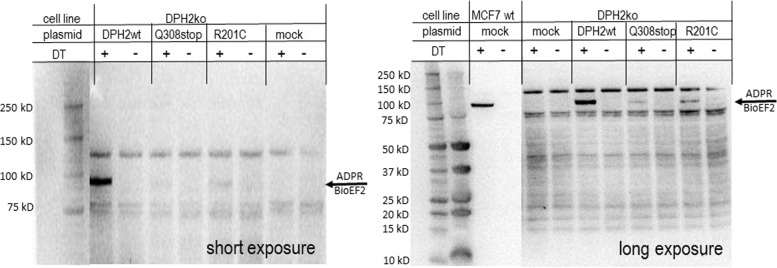
Toxin-mediated ADP-ribosylation (ADPR) assay. Cell extracts from MCF7 *DPH2*ko cells transfected with plasmids expressing DPH2 wt, c.922 C > T (p.(Gln308*) (Q308*)) or c.601 C > T (p.(Arg201Cys) (R201C)) variants were subjected to the ADPR activity of DT (+). Reactions without DT (−) and extracts of mock-transfected cells served as negative controls. Capability of cells to synthesize diphthamide is revealed by the generation of ADPR-eEF2 as a prominent band at ~100 kDA (indicated by red arrowheads). For this, *DPH2*ko cells transfected with wild-type *DPH2* serve as positive control, left panel: short exposure; right panel: long exposure.

The results of ADPR assays performed on extracts of DPH2-deficient MCF7 cells transfected with plasmids that encode either *DPH2* wild type or the *DPH2* variants are shown in Fig. 3. *DPH2*ko cells lack diphthamide-modified eEF2 as indicated by absence of bio-eEF2 signals in the ADPR-extracts of mock-transfected cells. Recombinant supplementation of wild-type *DPH2* rescues diphthamide synthesis defects in *DPH2*ko cells as shown by the presence of bio-eEF2 signals in the ADPR assay. In contrast, expression of the c.922C > T (p.(Gln308*)) and c.601C > T (p.(Arg201Cys)) variants generated barely any bio-eEF2 signals under otherwise identical ADPR assay conditions. Truncated DPH2 showed almost complete absence of diphthamide (just a very faint signal detectable upon long exposure). The c.601C > T (p.(Arg201Cys)) variant showed strongly reduced diphthamide signals (again unambiguously detectable only upon long exposure). Thus, both DPH2 variants presented with strongly reduced functionality.

### Analysis of yeast *DPH2* variants corresponding to c.922C > T (p.(Gln308*)) and c.601C > T (p.(Arg201Cys)) in hu*DPH2*

The components of the enzymatic machinery for diphthamide synthesis are highly conserved among eukaryotes including humans and yeast, and most studies on structure-function relations of diphthamide synthetic enzymes (Dph1–Dph7) so far have been performed in the budding yeast *S. cerevisiae* [1, 2]. Therefore, a lot of information related to residues affecting Dph2 protein activity is available for the yeast enzyme. This knowledge can be utilized to explain the molecular consequences of the disease-related mutations identified in huDPH2. Thus, we analyzed the effects of the c.922C > T (p.(Gln308*)) and c.601C > T (p.(Arg201Cys)) variants not only in human MCF7 cells but generated and analyzed yeast Dph2 variants with mutations corresponding to the huDPH2 variants. The positions in yeast Dph2 that correspond to c.922C > T (p.(Gln308*)) and c.601C > T (p.(Arg201Cys)) in huDPH2 were identified via alignment of the conserved primary sequences and protein structures (Fig. S1). Thus, the R201 position of huDPH2 was assigned to K239 of yeast Dph2 and the truncation (Q308) to K329. To analyze the effect of these variants on Dph2 activity, yeast genomic point mutations encoding the corresponding truncation or missense variant were generated using the *delitto perfetto* approach [20]. In contrast to plasmid-derived recombinant expression in human *DPH2*ko MCF7 cells described above, this approach assures that the yeast mutation is analyzed as single genomic copy controlled by its own promoter. Similar to the human c.922C > T (p.(Gln308*)) variant, yeast *dph2*K329* (note that human variants were called according to the HGVS format, yeast variants to *Saccharomyces* genome database guidelines) leads to the loss of a large part of the protein including one of the catalytic cysteines (C362) [27], and in analogy to the c.601C > T (p.(Arg201Cys)) variant in human DPH2, *dph2*K239C in yeast introduces an additional cysteine in proximity to the active site of Dph2. The above yeast mutant variants were analyzed in comparison to their respective wild-type (BY4741) cells and a *DPH2* deletion mutant (*dph2*Δ), which was shown to lack diphthamide on eEF2 [6, 10]. Thus, comparing the diphthamide synthesis capability of *dph2*Δ cells with *DPH2* wild type or mutant variants can address the enzymatic functionality of the enzymes in yeast.

**Fig. 4:**
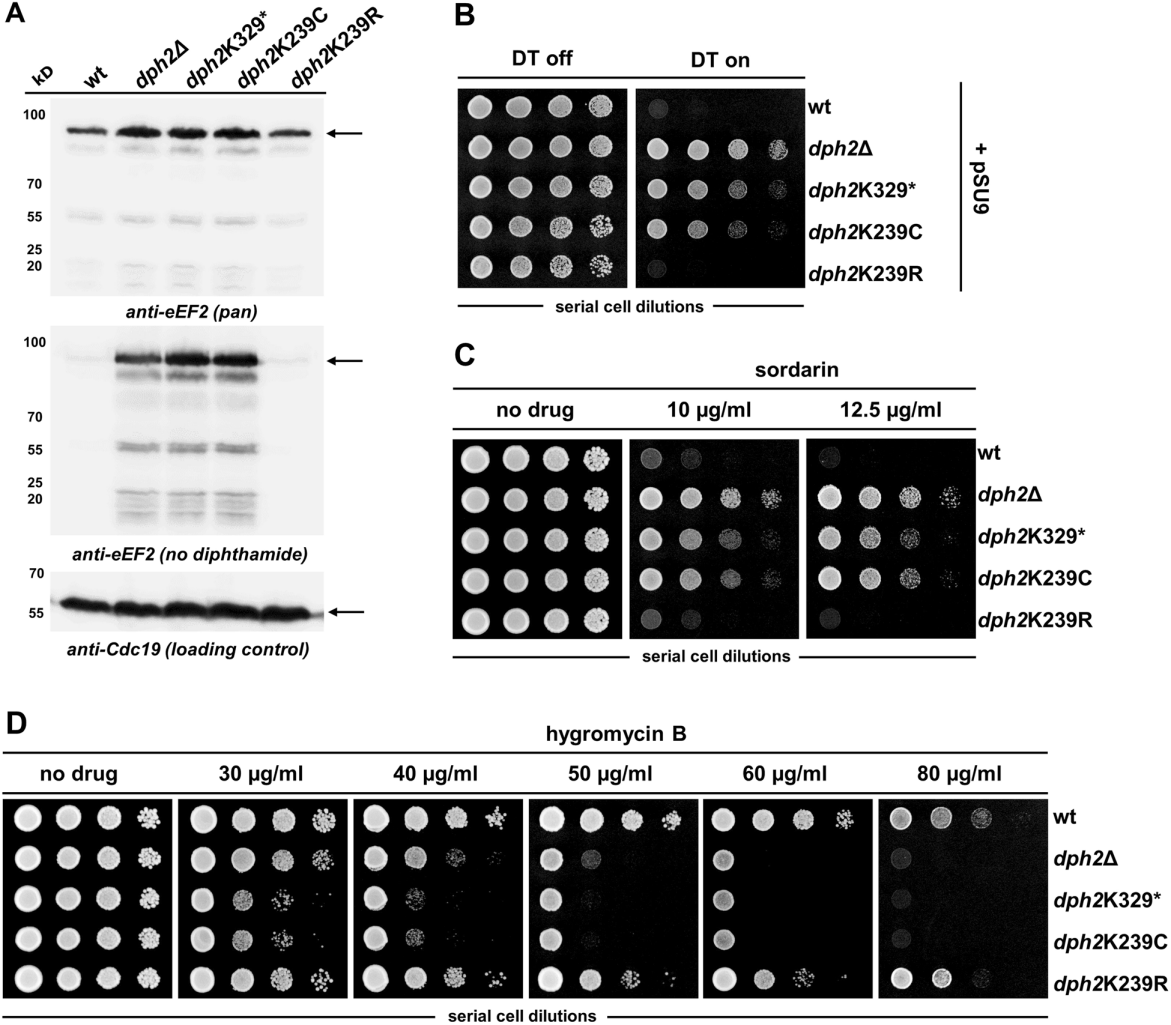
Analysis of yeast Dph2 variants. **a** Western blot to quantify total cellular eEF2 and unmodified eEF2 in several *dph2* mutants. Top panel: *Anti-eEF2(pan)* was used to detect eEF2 regardless of its modification status. Middle panel: *Anti-eEF2(no diphthamide)* specifically detects unmodified eEF2. Lower panel: to ensure equal loading, *anti-Cdc19* was used to detect pyruvate kinase Cdc19 used as loading control for protein extracts from yeast. **b** Resistance toward growth inhibition by the diphthamide-dependent diphtheria toxin (DT). Cells carrying the glucose-repressible and galactose-inducible DT expression vector pSU9 [5] were serially diluted and cultivated on medium containing glucose (DT expression: off) or galactose (DT expression: on) at 30 °C for 3 days. **c** Resistance to sordarin was determined by yeast cultivation in the presence of 10 and 12.5 µg/ml sordarin [29]. **d** Sensitivity to diphthamide-responsive translation inhibitor hygromycin B was assessed by cultivation on medium containing the indicated doses of hygromycin B.

To detect the presence or absence of diphthamide in yeast, two different approaches were used. Unmodified eEF2 in extracts of wild type and mutant cells was detected via Western blot (Fig. 4a) with an antibody that binds unmodified but not diphthamide or precursor modified eEF2 [8]. In addition, the presence/absence of diphthamide was detected by determining the cellular sensitivity (Table 2) to plasmid based, GAL promoter-driven expression of DT using pSU9 [5] or sordarin, to both of which diphthamide-deficient cells exhibit resistances [29]. In contrast, loss of diphthamide induces sensitivity toward transitional stress induced by hygromycin treatment [6]. The Western blots (Fig. 4a) show that the truncated Dph2 variant as well as the K239C mutant (reflecting c.601C > T (p.(Arg201Cys)) in huDPH2) maintain significant amounts of unmodified eEF2, similar in levels to *dph2*Δ cells. Thus, diphthamide synthesis is drastically reduced in yeast cells whose Dph2 activity is compromised by these mutations. In contrast, only small quantities of unmodified eEF2 were detected in cells expressing wt Dph2 or K239R which is consistent with proper Dph2 activity.

**Table 2:** Outcome of yeast viability assays to assess presence or absence of the diphthamide modification on eEF2.

*DPH2*	Viability of BY4741 cells			
	Medium	12.5 µg/ml sordarin	DT (recomb. induced)	80 µg/ml hygromycin
Wild type	+	−	−	+
K239R (control mut)	+	−	−	+
K239C	+	+	+	−
K329*	+	+	+	−
Deletion	+	+	+	−

Cell growth was analyzed on media containing sordarin, DT, and hygromycin (for details see Fig. 4b–d). + indicates cell growth; − indicates sensitivity/no cell growth.

The results of the phenotypic analyses in yeast are shown in Fig. 4b–d and summarized in Table 2. Dph2 wild type and one control mutant (K239R) were sensitive to DT (Fig. 4b) and to the applied concentrations of sordarin (Fig. 4c) and resistant to the applied concentrations of hygromycin (Fig. 4d), indicating diphthamide proficiency in these yeast strains. One positively charged residue (K) is exchanged with another (R) in the K239R mutant indicating minor changes at this position by substitution of comparable amino acids does not affect Dph2 activity. We consider this to be expected as an arginine residue is also present at the corresponding human position (R201). Hence, these lysine and arginine residues are interchangeable without functional impairment of yeast Dph2. In contrast, yeast cells that are Dph2 deficient (*dph2*Δ) or carry the K329* or K239C mutations were resistant to sordarin and DT, and hypersensitive to hygromycin (Fig. 4b–d). This indicates deficiency of diphthamide synthesis and confirms that those mutations render Dph2 inactive.

## Discussion

To our knowledge, we report here the first human phenotype associated with biallelic loss-of-function of *DPH2*, a human gene involved in diphthamide modification of eEF2 [27]. This conclusion is supported by two lines of evidence. First, biochemical studies of the *DPH2* variants in human and yeast cells are consistent with a loss-of-function of the DPH2 enzyme in this patient. Importantly, loss of diphthamide modification of eEF2 was documented in this case in vitro as was also observed for *DPH1* mutations found in patients with DPH1 syndrome. Second, reduced functionality of *DPH1*, also acting in the first step of the diphthamide biosynthesis pathway, leads to similar phenotypes as the patient presented here, including intellectual disability, prominent forehead, short stature, cardiac defects, and other dysmorphic features. Both macrocephaly and microcephaly have been described in individuals with variants in *DPH1* and this patient also presented with abnormal head circumference (macrocephaly).

As for the biochemical analysis of the alleles encoding mutated DPH2 proteins, we find that the DPH2 truncation and the missense variant enzymes both demonstrated dramatically reduced functionality. This is in contrast to the K239R mutation in yeast Dph2, which was generated as a control. The truncation (human c.922C > T (p.(Gln308*)) and corresponding yeast K329*) lacks a portion of the protein that contributes to the catalytic activity, including the cysteine C341 in human DPH2 (C362 in yeast Dph2; Figs. 2 and S1) which was previously indicated to be essential for Dph2 function in yeast, likely due to [4Fe–4S] cluster binding [27]. This explains that truncations at this position abolish (yeast assays) or strongly reduce (mammalian cell assays) DPH2 functionality (Figs. 3 and 4 and Table 2). In addition to deleterious effects of truncation or amino acid exchange, it is not unlikely that those alterations also affect protein structure, folding or stability and hence overall levels of DPH2 protein harboring those variants. Our assays to experimentally assess the human variants involve recombinant transient multicopy plasmid-based expression systems, such assays do not allow conclusions for expression levels in the relevant real-life chromosome-encoded settings. It is nevertheless reasonable to assume that reduced levels of these DPH2 variants may also contribute to or aggravate reduced functionality.

A truncated *DPH2* variant was previously described as a loss-of-function allele that can protect cells against *Pseudomonas* exotoxin A and DT [30]. The truncation displayed a dominant negative phenotype in the presence of functional *DPH2* alleles by preventing diphthamide modification of eEF2. Lack of clinical manifestations in the heterozygous parent that carries the truncation combined with a functional *DPH2* allele indicates that the identified disease-associated c.922C > T (p.(Gln308*)) truncation presents as inactive but not as dominant negative variant. Furthermore, the pLI score of 0 in gnomAD [25] indicates no depletion of heterozygous loss-of-function variants in the general population, also arguing against common dominant negative effects with this type of variant in humans. The DPH2 missense variant (human c.601C > T (p.(Arg201Cys)) and corresponding yeast K239C) also abolishes most enzyme activity (Figs. 3 and 4 and Table 2). This mutation results in an additional cysteine residue close to the active site of DPH2 (Figs. 2 and S1). The catalytic center of DPH2 is by itself composed of several optimally oriented cysteines that act as ligands for an iron–sulfur cluster co-factor (Fig. 2). Loss of function of the missense variant may reflect interference of the additional Cys-based thiol group (c.601C > T (p.(Arg201Cys)) or K239C) with the functionality of the Dph1–Dph2 complex [29, 31], and further details in Fig. S2. In contrast to most patients that defined the *DPH1*-syndrome, the *DPH2* patient described here has no consanguineous background but presents as a compound heterozygous genotype. One defective *DPH2* allele was inherited from each unaffected parent. Lack of a phenotype in individuals that still carry one functional *DPH2* gene is consistent with the observation that one intact copy of the *DPH1* or *DPH2* genes suffices to assure diphthamide synthesis on eEF2 in mammalian cells [8, 32]. Thus, there is no evidence for gene-dose/copy number associated phenotypes in individuals carrying one defective *DPH1* or *DPH2 *gene copy.

Until now, no similar or other disease/disability has been reported in the literature in association with *DPH2* gene deficiency with one possible exception. We propose that the siblings reported in 2016 [32] may in fact owe their phenotype to *DPH2* rathern than *KALRN*. Given our evidence for a very similar phenotype upon biallelic loss-of-function variants in *DPH2*, these patients may possibly represent additional unrelated cases, further supporting the association of *DPH2* loss-of-function variants with the phenotype reported here. To our knowledge, there have not been further reports of syndromic intellectual disability in individuals with biallelic *KALRN* variants. The GeneMatcher service (https://genematcher.org) was queried for *DPH2* in June 2019 with two matches: both involved heterozygous de novo missense variants that do not match the recessive inheritance model reported for *DPH1* and proposed here for *DPH2*. The verbally reported phenotypes did not clearly overlap with the case reported here.

The loss of function of different genes in the same biochemical pathway may lead to clinically indistinguishable phenotypes as has been observed in disorders of peroxisomal biogenesis (at least 13 genes) [33], Maple Syrup Urine Disease (OMIM #248600, 3 genes) [34], glycine encephalopathy (OMIM #605899, 3 genes) [35], and many others. For other biochemical pathways, phenotypes are distinct among defects at different steps in the same pathway, such as among different disorders of cholesterol biosynthesis, or deficiencies in the catabolism of tyrosine, mucopolysaccharides, or sphingolipids. It is therefore an important observation of this study that, at least between *DPH1* and *DPH2*, there is considerable phenotypic overlap, possibly united by the common failure to modify eEF2 with ACP, the first intermediate in diphthamide biosynthesis. Why the loss of eEF2-diphthamide modification leads to an intellectual disability syndrome is unclear. However, eEF2-diphthamide is essential for translation and hence may be considered a special type of ribosomopathy [36] or more widely as translation-associated deficiency [37]. Additional disorders of ribosome function include the Diamond–Blackfan anemias [38, 39] and Shwachman–Diamond syndrome [40] and lead to some overlapping congenital anomalies, such as heart and limb malformations and some dysmorphic features [41]. Those disorders are also associated with poor growth and developmental delay, but present with bone marrow dysfunction as a defining feature. Bowen–Conradi syndrome (OMIM #211180) [42], another ribosomopathy due to recessive variants in *EMG1* leading to defective ribosome biogenesis, is associated with severe developmental effects, poor growth, and physical anomalies, but interestingly not bone marrow failure. Why different ribosomopathies display such divergent phenotypes and mechanisms of inheritance is not known and may reflect the complexity of ribosome assembly and function. Interestingly, loss of a posttranscriptional tRNA anticodon modification required for proper mRNA decoding and proteostasis has been associated with human neuropathies and syndromes such as familial dysautonomia [43], intellectual disability [44], and amyotrophic lateral sclerosis [45]. These cases are linked to mutations in ELP1–ELP3, three subunits of the tRNA modifying Elongator complex. Hence, the above listed pathologies represent clinical cases with similarities to the diphthamide syndrome described here, potentially resulting from defects in translation elongation [46].

One explanation for the developmental phenotypes of diphthamide deficiency may be that it modulates stress- and development-related pathways and transcriptional patterns in mammalian cells. These include phosphorylation events involved in regulation of translation [47], responses to oxidative stress [24], NF-κB responses, and pathways including tumor necrosis factor-mediated pathways [8]. The latter processes, in particular NF-κB-associated transcriptional responses, are relevant for general as well as neuronal development [48]. The fact that animals with complete biallelic inactivation of *DPH* genes display embryonic lethal phenotypes with developmental defects provides further evidence for the relevance of diphthamide-modified eEF2 in development [14, 49, 50]. Embryonic lethality of animals with complete *DPH* gene inactivation may also explain why correlations between *DPH* gene defects and human disease have so far been observed only rarely. Compound heterozygous combinations or homozygous variants that completely abrogate diphthamide synthesis may be lethal. The notion that complete prevention of diphthamide synthesis may be embryonic lethal is also corroborated by the fact that all described *DPH1* patients [15] as well as the herewith described *DPH2* patient carry mutations or combinations that lead to reduced but not completely inactivated DPH1/DPH2 enzyme functions. The population frequency of variants in diphthamide synthesis genes that have sufficient function to avoid lethality but still result in a phenotype may be very low and explain the apparent rarity of disorders of diphthamide biosynthesis.

Additional screenings and analysis of populations and/or clinical cases will be required to confirm the association of *DPH2* with the phenotypes described here and to determine the full spectrum of phenotypes associated with this gene and indeed other steps in the diphthamide biosynthesis pathway that do not currently have associated human loss-of-function phenotypes. Because eEF2 is an essential nonredundant translation factor and diphthamide deficiency affects the accuracy of ribosomal protein biosynthesis, we propose that DPH1- and DPH2-related disease (and perhaps other components of this pathway) define a “diphthamide-deficiency syndrome” as a novel ribosomopathy inflicted by reduced functionality of components of the cellular eEF2-diphthamide synthesis machinery.

## Supplementary information

Supplemental Data
