# Supplementary material for: Diphthamide-deficiency syndrome: a novel human developmental disorder and ribosomopathy
Source: Eur J Hum Genet. 2020 Jun 23;28(11):1497–508. doi: 10.1038/s41431-020-0668-y (PMC7575589; doi:10.1038/s41431-020-0668-y)
Supplement: Supplementary file 1 — Supplemental Data [file 41431_2020_668_MOESM1_ESM.docx]

**Supplemental Data**

**Diphthamide-Deficiency Syndrome: A Novel Human Developmental Disorder and Ribosomopathy**

Harmen Hawer^1*^, Bryce A. Mendelsohn^2,6*^, Klaus Mayer^3^, Ann Kung^2^, Amit Malhotra^2^, Sari Tuupanen^4^, Jennifer Schleit^5^, Ulrich Brinkmann^3,6^, Raffael Schaffrath^1^

^1^ Fachgebiet Mikrobiologie, Institut für Biologie, Universität Kassel, Hessen, Kassel, D-34132, Germany.

^2^Kaiser Permanente Oakland Medical Center, CA, Oakland, 94611, USA.

^3^Roche Pharma Research & Early Development, Large Molecule Research, Roche Innovation Center Munich, Bavaria, Penzberg, D-82377, Germany.

^4^Blueprint Genetics Oy, Keilaranta 16 A-B 02150 Espoo, Finland ^5^Blueprint Genetics, WA, Seattle, 98121, USA.

^6^ Correspondence: Bryce.A.Mendelsohn@kp.org, ulrich.brinkmann@roche.com

* These authors contributed equally to this work


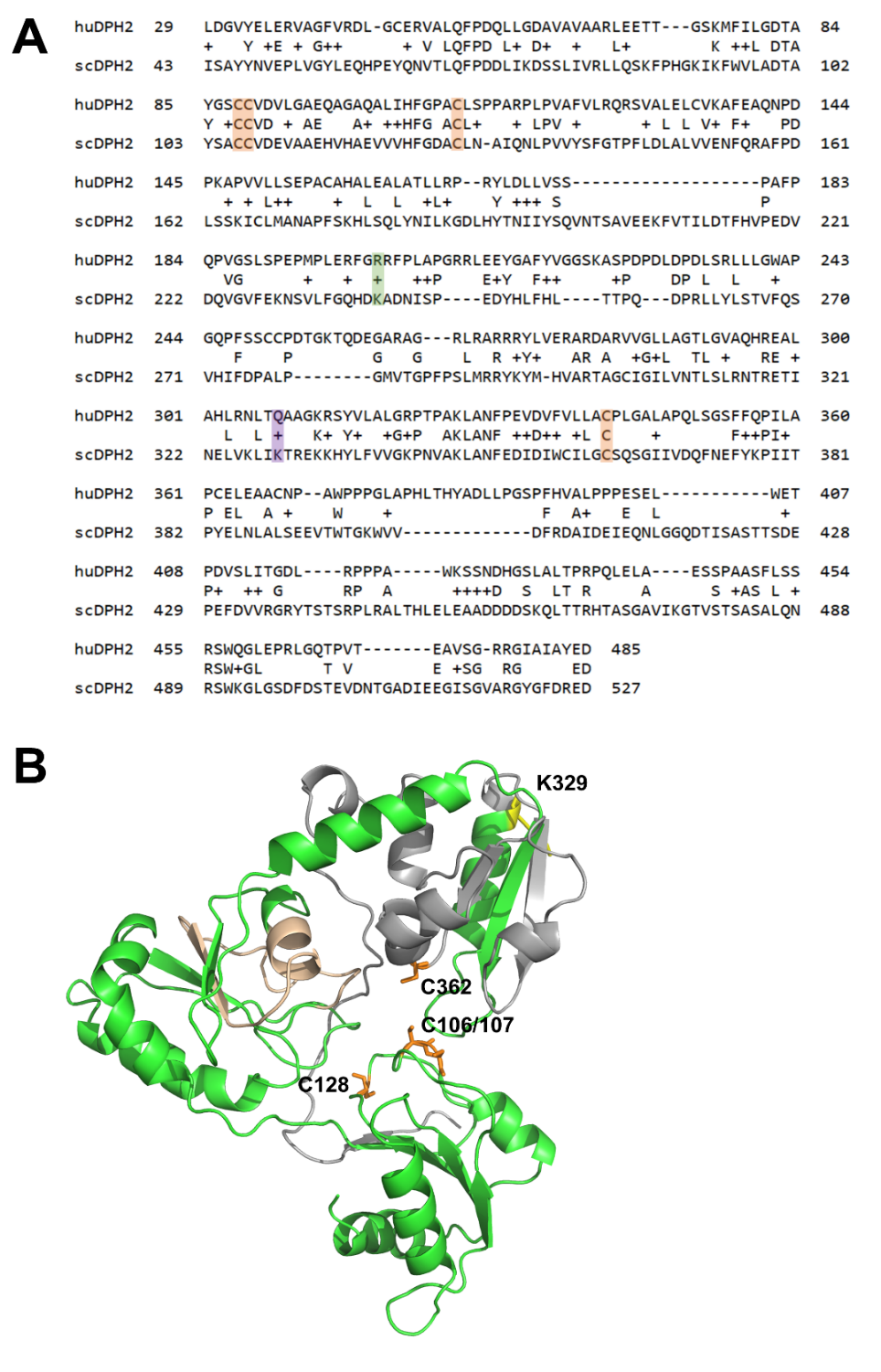


**Fig. S1** Sequence alignment and structure analysis of *Saccharomyces cerevisiae* Dph2 to visualize locations of mutations that correspond to c.922C>T (p.(Gln308*)) and c.601C>T (p.(Arg201Cys)) variants in the human counterpart (huDPH2). **A**. Amino acid sequence alignment of human DPH2 and yeast Dph2. Orange: conserved cysteine residues. Green: Position of R201 and K239 in human and yeast DPH2, respectively. Violet: Position that marks the location of the truncating variant in the patient (c.922C>T (p.(Gln308*)) as well as the assigned yeast position (K329*****). Alignment was calculated with protein BLAST and standard algorithm parameters using human DPH2 as query and *S. cerevisiae* Dph2 as subject. Analysis revealed a 93 % query cover. **B**. Structural model of yeast Dph2. The model shown derives from alignment with the template structure of Dhp2 from *Pyrococcus* (PDB:3LZD, ([1](#_ENREF_1))). Orange: Structure alignments identify cysteines at positions 106/107 and 362 to be homologous to two of the cysteines (C59 and C287) that bind the [4Fe-4S] cluster in *Pyrococcus* Dph2. An additional cysteine is located at position 128. The truncation mutation (K329*****) corresponding to the one found in the patient c.922C>T (p.(Gln308*)) is highlighted in yellow. The region containing K239 is highlighted in wheat.


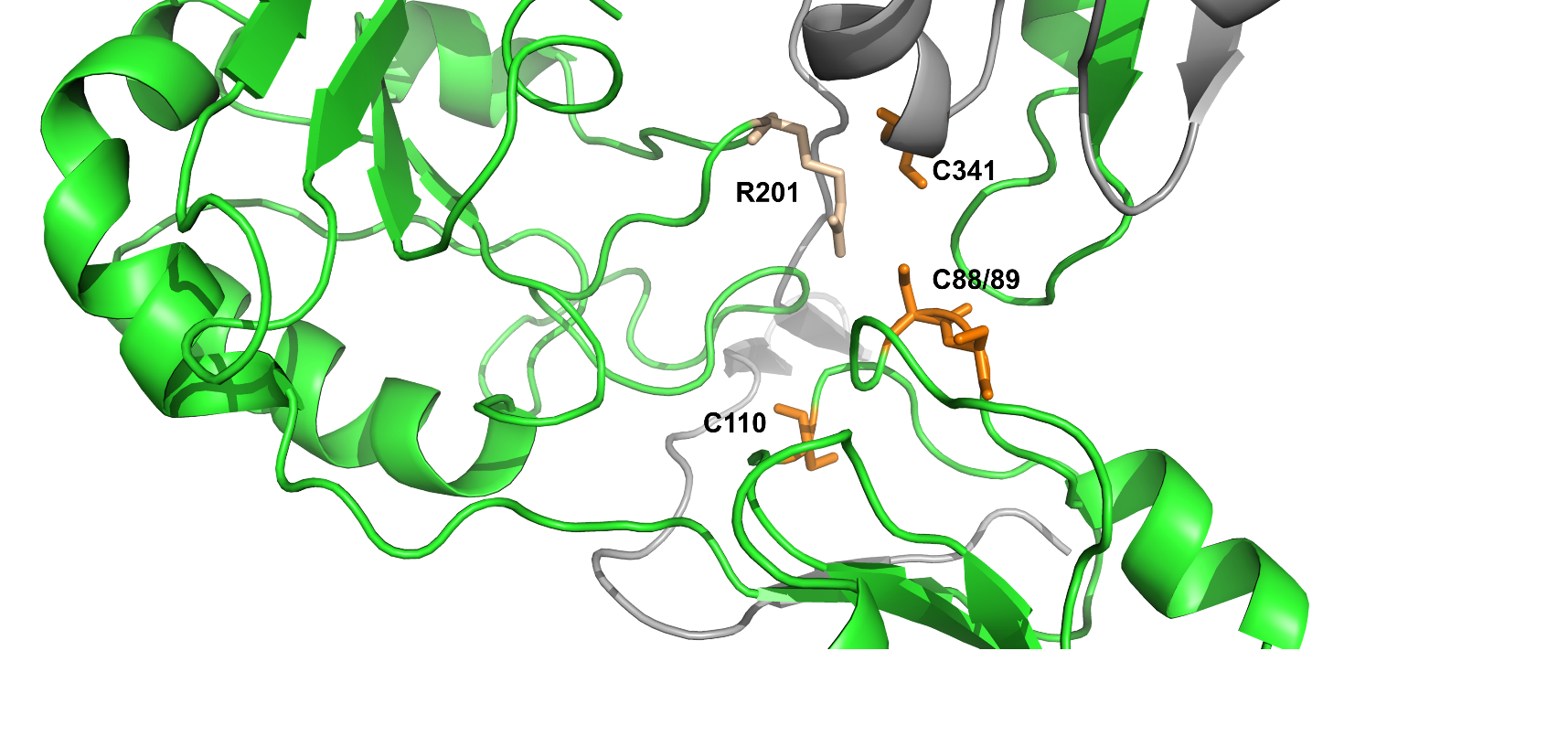


**Fig. S2** Structural model of human DPH2 showing the c.601C>T (p.(Arg201Cys)) variant places an additional cysteine in close proximity to the active center. Orange: Cysteines at positions 88/89, 110 and 341. Point mutated residue found in the patient (R201) highlighted in wheat. Grey: Region that is predicted to be absent in the c.922C>T (p.(Gln308*)) truncation variant. The additional cysteine introduced by c.601C>T (p.(Arg201Cys)) in human DPH2 may affect functionality because it either destabilizes the catalytic center and the overall structural integrity of the protein, or impairs the binding of the iron sulfur cluster co-factor and interferes with the electron transfer cascade essential for the catalytic activity of the DPH1-DPH2 heterodimer.

**References**

1. Zhang Y, Zhu X, Torelli AT, Lee M, Dzikovski B, Koralewski RM, et al. Diphthamide biosynthesis requires an organic radical generated by an iron-sulphur enzyme. Nature. 2010;465(7300):891-6.
